# Philosophical Transactions of the Royal Society of London

**Published:** 1783

**Authors:** 


### THE LONDON MEDICAL JOURNAL,

For JANUARY, FEB. and MARCH,
1783.

### SECTION I.

### BOOKS.

I. *Philosophical Transactions of the Royal Society of London. Vol.* LXXII. *for the year* 1782. *Part* I. 4to. London, 1782. 337 pages, with 6 copper-plates.

1. *NOVA Experimenta Chemica quæ ad penitiorem acidi e pinguedine eruti cognitionem valere videntur. Scribebat D.* Laurentius Crellius, Gulielmo Huntero, M. D. S. R. S.——Professor Crell in this paper continues his inquiries into the chemical properties of the acid obtainable from animal fat. Sixty experiments are related, for an account of which we must refer the chemical reader to the work itself.

II. *Observations on the Bills of Mortality at York. By* William White, M. D. F. A. S.

From a table of births and burials in York, from the year 1728 to 1735, inclusive, published by Mr. Drake in his Antiquities of York, it appears that during that period the proportion of deaths was 1 in 21$\frac{3}{4}$. "Very different from "this—says the author of the paper before us— "is our present situation, the proportion of "deaths being now decreased to 1 in 28$\frac{1}{4}$, "which is the quotient of 12,800, the number "of inhabitants, divided by 453, the present "average of annual deaths. This is certainly "a great rise in the scale of healthiness. From "being near as fatal as London, we have become "less so than many country places, as will ap- "pear from the following comparative view of "the proportion of deaths in different places:

| | | |
|---|---|---|
| "At Vienna, | - | 1 in 19$\frac{1}{2}$ dies every year. |
| London, | - | 1 in 20$\frac{3}{4}$ |
| Edinburgh, | - | 1 in 20$\frac{3}{4}$ |
| Berlin, | - - | 1 in 21 |
| Rome, | - - | 1 in 22 |
| Amsterdam, | - | 1 in 22 |
| Dublin, | - - | 1 in 22 |
| Leeds, | - - | 1 in 22 |
| Northampton, | | 1 in 26 |

"At

| | | | |
|---|---|---|---|
| " At Shrewſbury, | - | 1 in 26 | dies every year. |
| Liverpool, | - | 1 in $27\frac{7}{10}$ | |
| Mancheſter, | - | 1 in 28 | |
| York, | - - | 1 in $28\frac{1}{4}$" | |

It appears from Dr. White's obſervations, that the ſummer ſeaſon is by much the healthieſt at York; autumn the next; then the ſpring; winter being by far the moſt fatal. Dr. Percival found much the ſame to be the caſe at Mancheſter. At Cheſter, Dr. Haygarth ſays, November was the moſt ſickly month.

The diſeaſes at York, we are told, are chiefly of the inflammatory kind, which are known to be the general attendants of the winter and ſpring months.

Among the general cauſes of the increaſing population and healthineſs of York, Dr. White enumerates the introduction of inoculation, improvements in the treatment of ſeveral diſorders, the cool regimen in fevers, the admiſſion of freſh air, and the general uſe of antiſeptic medicines and diet. To theſe, he thinks, may be added a general improvement and greater attention to Nature in the management of infants.

After the general cauſes of healthineſs, ſuch as are particular, or of a more local nature, come under our author's conſideration. In this respect, it seems, the city of York has been much improved within a few years past. The streets have been widened in many places, by taking down a number of old houses, built in such a manner as almost to meet in the upper stories, by which the sun and air were almost excluded in the streets and inferior apartments. They have also been new paved, additional drains made, and, by the present method of conducting the rain from the houses, are become much drier and cleaner than formerly. The erection of the locks, about four miles below the city, has been a great advantage to it: for, before this, the river was frequently very low, leaving quantities of dirt in the very heart of the city, also the filth of the common sewers, which it was unable to wash away. The lock has effectually prevented this for the future, by the river being kept always high, broad, and spacious; and has thus contributed to the salubrity as well as beauty of York.

III. *Account of a Monstrous Birth. In a letter from* John Torlese, *Esq. Chief of Anjingo, to the Hon.* William Hornbey, *Governor of Bombay. Communicated by Dr.* Lind, F. R. S.——A Nair woman was delivered at Anjingo of the child here described, on the 28th of March 1780, and and it lived, we are told, till the 1ſt of April in the morning. In the afternoon Mr. Torleſe went to ſee it. His account of it is as follows: "It had but one body, at the extremity whereof "were two heads, one larger than the other. "It had four hands and arms perfect, two legs "on one ſide its body, and one on the other, "which began on the middle of its back, and "appeared by Nature intended for two by its "ſize, and from the appearance of the foot, "which looked as if two had been ſqueezed "or rather maſhed together. It had only one "navel, and one anus, but two genitals of the "female. It was fed, during its ſhort exiſtence, "by hand, with goats milk. It is remarkable, "that one head would ſleep whilſt the other "was awake; or one would cry, and the other "not. They both died at the ſame inſtant. "Almoſt all this town went to ſee it, the like "having never been heard of before. The "mother is a ſtout woman; and I ſaw four of "her children at her houſe, the youngeſt of "which was ſix years old, all healthy and per-"fect." Two engravings are given of this monſter.

IV. *Continuation of the Experiments and Obſervations on the ſpecific Gravities and attractive*

*Powers*

*Powers of various Saline Subſtances* *. *By* Richard Kirwan, *Eſq*. F. R. S.——In this paper the learned and indefatigable author proceeds to examine the quantity of pure acids taken up at the point of ſaturation by the various ſubſtances they unite with. His experiments are arranged under the following heads; viz. of the mineral alkali;—volatile alkali;—calcareous earth;—magneſia, or muriatic earth;—earth of alum, or argillaceous earth;—phlogiſton;—quantity of phlogiſton in nitrous air, fixed air, vitriolic air, ſulphur, and marine acid air.

In treating of phlogiſton, the ingenious author begins with explaining the origin and formation of inflammable air; after which he proceeds to ſhew its identity and homogeneity with phlogiſton. By phlogiſton is generally underſtood that principle in combuſtible bodies on which their inflammability principally depends; that principle to which metals owe their malleability and ſplendor; that which, combined with vitriolic acid, forms ſulphur; that which diminiſhes reſpirable air. Inflammable air, according to Mr. Kirwan, is that very principle which alone is truly inflammable. He preſents us with a

* See the 2d vol. of this Journal, p. 191.

great great number of obſervations and experiments from himſelf and others, but particularly from Dr. Prieſtley, in ſupport of this doctrine. From theſe he thinks it ſufficiently proved "that in-"flammable air, purified from the acids or "other ſubſtances that expel it from its baſis, "and alſo from all particles of the body to "which it was originally united, ſuch as in-"flammable air from metals received on mer-"cury, and well waſhed in lime water, is one "and the ſame ſubſtance with phlogiſton, dif-"fering only in quantity of fire, inflammable "air containing nearly the ſame quantity of this "element as the ſame bulk of atmoſpheric air, "as Dr. Crawford has found by ſome late ex-"periments, an account of which will ſoon be "laid before the public."

It may appear extraordinary, ſuppoſing inflammable air and phlogiſton to be the ſame ſubſtance, that inflammable air ſhould mix ſo eaſily with water, whereas phlogiſton conſtantly repels and is repelled by it; but this, we are told, depends on the ſtate of this ſame ſubſtance, which when fixed and concrete, is called *phlogiſton*, and when rarefied and aëriform, *inflammable air*. In this latter ſtate, Mr. Kirwan obſerves, it mixes with water in proportion to its rarefaction.

The

The other papers contained in this volume are, *Relazione di una nuova Pioggia, ſcritta dal Conte de* Gioeni, *abitante della 3a reggione dell' Etna.* i. e. *Account of a new kind of Rain. Written by the Count de* Gioeni, *an inhabitant of the 3d region of Mount Etna.*——*Experiments with Chineſe Hemp-ſeed. By* Keane Fitzgerald, *Eſq.*——*An Account of ſome Scoria from Iron works, which reſemble the vitrified filaments deſcribed by Sir* William Hamilton. *By* Samuel More, *Eſq.*——*An Abſtract of the Regiſter of the Pariſh of Holy Croſs, Salop,* from 1770 to 1780. *By the Rev.* William Gorſuch, *Vicar.*——*An Experiment propoſed for determining, by the aberration of the fixed ſtars, whether the rays of light, in pervading different media, change their velocity according to the law which reſults from Sir* Iſaac Newton'*s ideas concerning the cauſe of refraction; and for aſcertaining their velocity in every medium whoſe refractive denſity is known. By* Patrick Wilſon, M. A.——*Quantity of Rain which fell at Barrowby near Leeds. By* George Lloyd, *Eſq.* F. R. S.——*An Account of an improved Thermometer. By Mr.* James Six.——*Of the Parallax of the Fixed Stars. By Mr.* Herſchel, F. R. S.—*Catalogue of Double Stars. By* the ſame.—*Deſcription of a Lamp Micrometer. By* the ſame.—*A Paper*

*to*

*to obviate ſome doubts concerning the great magnifying powers uſed.* *By* the ſame.—*Del modo di render ſenſibiliſſima la più debole Elettricità ſia naturale, ſia artificiale.* i. e. *Of the method of rendering very ſenſible the weakeſt natural or artificial electricity. By Mr.* Alexander Volta, *Profeſſor of Experimental Philoſophy in Como.*——*Abſtract of a Regiſter of the Barometer, Thermometer, and Rain, at Lyndon in Rutland,* 1780. *By* Thomas Barker, *Eſq.*——*Meteorological Journal kept at the houſe of the Royal Society.* This is brought down no lower than the month of Auguſt; and we are ſorry to learn that the Society, for want of proper convenience in their new apartments in Somerſet Place, have been obliged to diſcontinue their meteorological obſervations.

